# Limit of Physician’s Responsibility

**Published:** 1875-04

**Authors:** 


The Limits of the Physician's Responsibility.—In the recent suit of Rankin *vs.* McCarrell, for alleged malpractice, in the Common Pleas, Pittsburgh, Judge Ewing charged the jury as follows:

"The defendant, as a physician, if he understood the case of this boy, was bound to have and exercise the ordinary knowledge, skill and care of his profession, having a regard to the advanced state of the science at the time. If he failed in this, and injury resulted to the boy therefrom, the defendant is liable. But if he had and exercised this ordinary knowledge, skill and care, it was all that he was required to do, and it matters not what was the final result of the accident or disease of the boy; it is his misfortune; he cannot recover from his physician. A physician does not and cannot be expected to secure the recovery of his patients. He deals with material very different from that upon which mechanics operate. His business is with muscles, and nerves, and joints, and organisms, fully understood only by Omniscience. With a high respect for the medical profession, their science is an uncertain one. All that any one of the most skilled and learned can do is to exercise his best skill and judgment in the particular case, and when he has done that, if he has ordinary knowledge and skill, he has done all that can be required of him."—*Medical and Surgical Reporter.*

